# Better documentation in electronic medical records would lead to an increased use of lower extremity venous ultrasound in the inpatient setting: a retrospective study

**DOI:** 10.1002/ams2.289

**Published:** 2017-06-19

**Authors:** Daisuke Takechi, Naoto Kuroda, Hisashi Dote, Euido Kim, Osamu Yonekawa, Takuya Watanabe, Tetsumei Urano, Yoichiro Homma

**Affiliations:** ^1^ Department of General Internal Medicine Seirei Hamamatsu General Hospital Hamamatsu Shizuoka Japan; ^2^ Department of Emergency & Critical Care Medicine Seirei Hamamatsu General Hospital Hamamatsu Shizuoka Japan; ^3^ Department of Laboratory Medicine Seirei Hamamatsu General Hospital Hamamatsu Shizuoka Japan; ^4^ Department of Physiology Hamamatsu University School of Medicine Hamamatsu Shizuoka Japan

**Keywords:** copy and paste, D‐dimer, electronic medical record, Health communication, Joint Committee International

## Abstract

**Aim:**

We hypothesized that the quality of the assessment of abnormal laboratory data in the emergency department (ED) could affect the hospital‐attending physicians’ decision‐making after a patient's hospitalization. To test this hypothesis, we investigated how patients with a positive D‐dimer result were reported by ED physicians in electronic medical records, and measured whether lower extremity venous ultrasonography examination was undertaken during hospitalization by the hospital‐attending physicians.

**Methods:**

In an urban tertiary acute care general hospital in Japan, between January 2012 and December 2013, we included patients hospitalized after a positive D‐dimer measurement (≥1.0 μg/mL) that was taken in the emergency department. We retrospectively measured the quality of ED physician assessments. Then we examined whether that affected the decisions of attending physicians to order lower extremity venous ultrasonography examinations during hospitalization. The exposure variable was the quality of the ED physicians’ assessment of patients with positive D‐dimer results. The outcome was whether a lower extremity venous ultrasonography examination was ordered by the attending physician during hospitalization.

**Results:**

When assessments were described by ED physicians for patients with positive D‐dimer results, the attending physicians frequently ordered lower extremity venous ultrasonography (odds ratio, 10.74; 95% confidence interval, 5.92–19.50), even if the assessments only contained “copied and pasted” laboratory data (odds ratio, 1.68; 95% confidence interval, 2.10–2.40).

**Conclusions:**

Better documentation by ED physicians, regarding patients with positive D‐dimer results, strongly affected the decisions made by attending physicians to order lower extremity venous ultrasonography examination.

## Introduction

Computerized documentation is the direct entry of a physicians’ notes into the electronic medical record (EMR) through the intrahospital network, and offers improved legibility and real‐time accessibility.^1^ Such integrated EMR systems can ensure that a physician's documentation is easier to browse than a traditional paper record; thereby, providing reference for the next examination.^2^

The EMR system also offers time‐saving functions for potentially cumbersome and time‐consuming tasks such as the copy and paste function. Several studies have researched this copy and paste function. In fact, the copy and paste function has been considered a serious problem with the use of EMRs.^3^, ^4^, ^5^

However, none investigated the influence of the quality of documentation within EMRs in a cohort setting, even retrospectively. We hypothesized that the quality of the documentations of abnormal laboratory data in the emergency department (ED) could affect the hospital‐attending physicians’ decision‐making after hospitalization. To test this hypothesis, we investigated how patients with a positive D‐dimer result were reported by emergency physicians in the EMRs. Then we examined whether that affected the decisions of attending physicians to order lower extremity venous ultrasonography examination (venous US) during hospitalization.

## Methods

### Selection criteria

The study was undertaken at the ED of Seirei Hamamatsu General Hospital (Hamamatsu, Japan) using EMRs between January 2012 and December 2013. The hospital is an urban tertiary acute care general hospital with 744 beds, and annually, approximately 20,000 patients visit.

We included adult patients (over 18 years old) admitted from the ED after obtaining a positive D‐dimer result (D‐dimer ≥1.0 μg/mL). We excluded patients who had already been diagnosed with deep vein thrombosis (DVT), pulmonary embolism, aortic dissection, or cardiopulmonary arrest in the ED. The study was carried out in accordance with the Declaration of Helsinki, and the protocol was approved by the Institutional Review Board of Seirei Hamamatsu General Hospital.

### Data extraction of patient demographics

The following demographic data were collected: age, sex, primary disease, D‐dimer values, and death during hospitalization. We created the datasets by reviewing the EMRs of patients. This data was double‐checked by two of the authors. Primary disease was classified into four categories: cancer bearing,^6^ infection,^7^ trauma,^8^ and others. These primary diseases were considered to affect the ED assessment because each is associated with positive D‐dimer results.^6^, ^7^, ^8^ Death during hospitalization was considered to indicate a severe disorder and was treated as a confounder for both variables and outcomes.

In our area, five institutions are responsible for emergency duty on a rotating schedule. Therefore, we also collected information about admission at emergency duty days because these tend to be busy and may affect the assessments of emergency physicians. We did not collect the history of oral contraception use because its rate is very low in Japan.^9^

### Data extraction of exposure group/non‐exposed group

We classified the quality of documentation of positive D‐dimer results in the EMRs by emergency physicians into the following three groups: (i) ND group, no description or assessment of the positive D‐dimer result; (ii) CAP group, copied and pasted D‐dimer results (i.e., described only); (iii) LDD group, a listed differential diagnosis was provided for the positive D‐dimer.

In the differential diagnosis for the positive D‐dimer, we defined D‐dimer‐related diseases as DVT or pulmonary embolism,^6^, ^10^ aortic dissection,^11^ disseminated intravascular coagulopathy,^12^ and others. Documentation data were collected in a sentence, and two authors, D.T. and Y.H., discussed and classified them into three groups.

### Outcome measures

To evaluate whether the information was effectively transmitted from the emergency physicians to the hospital‐attending physicians through the EMRs, we determined whether venous US was carried out during the hospitalization. Venous US is the gold standard for the diagnosis of DVT, requiring well‐trained staff.^10^ All venous US were undertaken during the hospitalization in our institution because there were no suitably trained staff in the ED. As covariates, we selected age, sex, primary disease, D‐dimer values, and death during the hospitalization to adjust outcomes in this model.

Moreover, we observed when venous US was carried out during the hospitalization. Because long‐term admission may be related to DVT, we also measured the period (in days) from admission to when venous US was performed.

### Venous US and D‐dimer evaluation

D‐dimer was measured with a full‐automatic blood coagulation measuring device Sysmex CA‐1500 (Sysmex, Kobe, Japan) using the recommended reagent. The cut‐off value for a positive D‐dimer on this system was ≥1.0 μg/mL.

We defined all lower extremity venous thrombosis diagnosed by venous US as the onset of all DVT from the proximal to the distal calf. We chose this definition because calf DVT was also associated with a significant risk of subsequent post‐thrombotic syndrome.^13^ The venous USs were carried out by six laboratory technicians only during the daytime. We used LOGIQ ultrasound systems with 9‐MHz linear probes (GE Healthcare Japan, Tokyo, Japan). The clinical laboratory physician (O.Y.) controlled the quality of all examinations.

### Electronic medical record system and hospitalization

We used the MegaOakHR R5.0 (NEC, Tokyo, Japan) EMR system. The same EMR system is used in both the ED and during hospitalization. Therefore, hospital‐attending physicians can easily refer to medical records input by ED physicians.

Our hospital has been certified as an international standard hospital by the Joint Commission International since 2012. One of the certification requirements of the Joint Commission International is for completed documentation in the clinical records within the first 24 h of hospital admission. Therefore, the hospital‐attending physicians in the hospital should review the EMR assessments by emergency physicians within 24 h. The emergency physicians were always different from the hospital physicians because continuous work after ED duty was generally prohibited at our institution.

### Statistical analysis

We undertook statistical analyses using SAS version 9.3 (SAS Institute, Cary, NC, USA). Logistic regression analyses with corresponding odds ratios (OR) and 95% confidence interval (95% CI) were applied to adjust the covariates.

We undertook a pilot study with approximately 100 patients to facilitate the sample‐size calculation. We calculated the number of cases required identifying this 7.3% difference using the χ^2^‐test with α and β error levels of 0.05 and 0.2, respectively. The ratio of the exposure to the non‐exposed group was 0.20; therefore, approximately 80% of cases were in the exposure group, and 1,178 cases were required to calculate the predicted difference.^14^ Consequently, we set the research period to 2 years, as this allowed for seasonal variability in disease prevalence.^15^

## Results

### Patient demographics

**Figure 1:**
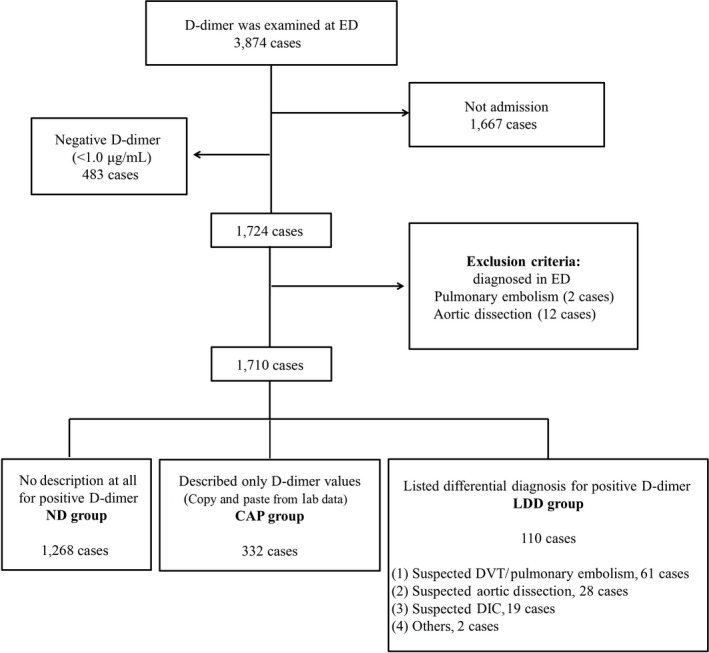
Patient distribution in the study of the influence of the standard of documentation in electronic medical records on the use of lower extremity venous ultrasound in the inpatient setting. DIC, disseminated intravascular coagulopathy; DVT, deep venous thrombosis; ED, emergency department.

D‐DIMER EXAMINATIONS WERE carried out in 3,874 patients during the study period in the ED of the hospital (Fig. 1). Of these, 1,667 were treated in the ED and released, and 2,207 were hospitalized. A further 483 had negative D‐dimer results (<1.0 μg/mL). Thus, 1,724 patients were enrolled in the study. We excluded 16 patients diagnosed in the ED (2 with pulmonary embolism and 14 with aortic dissection), leaving 1,710 patients for the final analysis set (Table 1). These patients were classified based on the quality of assessment, as follows: ND group (1,268 cases, 74.15%), CAP group (332 cases, 19.42%), and LDD group (110 cases, 6.43%). Among the 1,710 patients, 305 patients (17.8%) underwent venous US during their hospitalization. The length of time from admission to venous US was not significantly different between the three groups (Tukey–Kramer method, *P* = 0.71).

**Table 1:** Demographics of patients hospitalized after a positive D‐dimer measurement ( ≥1.0 μg/mL) that was taken in the emergency department (ED), grouped according to the quality of documentation in the electronic medical record

	Total	Quality of documentation in the ED		
		ND group	CAP group	LDD group
	1,710 cases	Cases (%)	Cases (%)	Cases (%)
		1,268 (74.15)	332 (19.42)	110 (6.43)
Sex				
Female	782	584 (74.68)	153 (19.57)	45 (5.75)
Male	928	684 (73.71)	179 (19.29)	65 (7.00)
Age, years				
<80	967	727 (75.18)	184 (19.03)	56 (5.74)
≥80	743	541 (72.81)	148 (19.92)	54 (7.27)
D‐dimer, μg/mL				
1.0 ≤ D‐dimer < 3.0	716	547 (76.40)	143 (19.97)	26 (3.67)
3.0 ≤ D‐dimer < 10.0	556	409 (73.56)	108 (19.42)	39 (7.02)
10.0 ≤ D‐dimer	438	312 (71.23)	81 (18.49)	45 (10.28)
Admission on emergency duty day^a^ (cases)	998	739 (73.48)	194 (20.79)	65 (5.73)
Death on hospitalization (cases)	279	205 (73.48)	58 (20.79)	16 (5.73)
Primary disease^b^				
Cancer bearing	199	160 (80.40)	32 (16.08)	7 (3.52)
Infection	660	464 (70.30)	144 (21.82)	52 (7.88)
Trauma	343	290 (84.55)	43 (12.54)	10 (2.91)
Others	591	424 (71.74)	123 (20.81)	44 (7.45)
Venous US performed^c^	305	192 (62.95)	64 (20.98)	49 (10.07)
DVT‐detected cases	61	25 (40.98)	22 (36.07)	14 (22.65)
From admission to venous US, days^d^	5.66 (±10.20)	5.91 (±0.84)	4.39 (±1.47)	5.90 (±0.83)

a Admitted on emergency duty day.

b Multiple chosen.

c During the patient's hospitalization period.

d Measured from the day of admission to the day at which venous ultrasound (US) carried out. No difference in these periods was evident between the three groups (two‐sided Tukey–Kramer method, *P* = 0.71).

CAP group, D‐dimer results copied and pasted (i.e., described only); DVT, deep venous thrombosis; LDD group, a listed differential diagnosis was provided for the positive D‐dimer; ND group, no description or assessment of the positive D‐dimer result; venous US, lower extremity venous ultrasonography examination during the patients’ hospitalization.

### Factors influencing documentation for positive D‐dimer assessment in the ED

First, the multivariate logistic analyses were carried out to examine the association of exposure (value of D‐dimer) with the outcome (CAP or LDD documentation) by adjusting for age, sex, death on hospitalization, and primary disease (Table 2). For trauma patients, neither CAP nor LDD documentation was prepared in the ED with ORs of 0.53 (95% CI, 0.36–0.77) and 0.29 (95% CI, 0.14–0.61), respectively. For patients who died during hospitalization, LDD documentation was associated with an OR of 0.44 (95% CI, 0.24–0.83), which was lower compared with 1.04 (95% CI, 0.74–1.50) for the CAP group.

**Table 2:** Factors influencing positive D‐dimer assessments in the emergency department

	CAP group 332 cases		LDD group 110 cases	
	Odds ratio	*P*‐value	Odds ratio	*P*‐value
Sex				
Female	1.00 (Ref.)		1.00 (Ref.)	
Male	0.97 (0.76–1.24)	0.800	1.32 (0.85–2.04)	0.2200
Age, years				
<80	1.00 (Ref.)		1.00 (Ref.)	
≥80	1.01 (0.79–1.30)	0.940	1.17 (0.76–1.80)	0.4800
Admission on emergency duty day				
No	1.00 (Ref.)		1.00 (Ref.)	
Yes	0.99 (0.77–1.26)	0.920	0.99 (0.64–1.52)	0.9500
Death in hospital				
No	1.00 (Ref.)		1.00 (Ref.)	
Yes	1.04 (0.74–1.50)	0.810	0.44 (0.24–0.83)	0.0110*
Primary disease†				
Cancer bearing	0.71 (0.47–1.07)	0.100	0.45 (0.19–1.04)	0.0620
Infection	1.07 (0.82–1.38)	0.620	1.11 (0.71–1.73)	0.6400
Trauma	0.53 (0.36–0.77)	0.001*	0.29 (0.14–0.61)	0.0010*
Others	1.00 (Ref.)		1.00 (Ref.)	
D‐dimer‐related disease				
No onset	1.00 (Ref.)		1.00 (Ref.)	
Onset during admission	1.32 (0.85–2.03)	0.210	11.98 (7.43–19.30)	2.0×10^−24^ *
D‐dimer, μg/mL				
1.0 ≤ D‐dimer < 3.0	1.00 (Ref.)		1.00 (Ref.)	
3.0 ≤ D‐dimer < 10.0	0.98 (0.74–1.31)	0.910	1.94 (1.13–3.32)	0.0160*
10.0 ≤ D‐dimer	1.03 (0.73–1.44)	0.880	2.60 (1.45–4.67)	0.0013*

^†^Multiple chosen.

The multivariate analysis was carried out using logistic regression analysis. *Two‐sided *P*‐values <0.05.

CAP group, described only D‐dimer values (copied and pasted from laboratory data); LDD group, listed differential diagnosis for positive D‐dimer; Ref.., reference.

D‐dimer values did not affect CAP documentation, with ORs of 0.98 (95% CI, 0.74–1.31) and 1.03 (95% CI, 0.73–1.44). However, higher D‐dimer values did affect LDD documentation, with ORs of 1.94 (95% CI, 1.13–3.32) and 2.60 (95% CI, 1.45–4.67) for D‐dimer values of 3.0–10.0 and >10.0, respectively.

### Factors that influenced venous US requests during hospitalization

To test our hypothesis, we compered the number of patients who underwent venous US among the ND, CAP, and LDD groups with the same D‐dimer level (Table 3). As the D‐dimer level increased, of course, venous US was carried out more often.

**Table 3:** Number of patients who underwent venous ultrasound (US) in hospital after recording the same positive D‐dimer measurement in the emergency department (ED), grouped according to the quality of documentation in the electronic medical record

	Total	Venous US performed		Quality of documentation in the ED			
				ND group	CAP group	LDD group	*P*‐value^a^
D‐dimer, μg/mL	1,710 cases			1,268 cases	332 cases	110 cases	
1.0 ≤ D‐dimer < 3.0	716	Yes	64	43	16	5	0.0800
		No	652	504	127	21	
3.0 ≤ D‐dimer < 10.0	556	Yes	105	65	17	23	<0.0010
		No	451	344	91	16	
10.0 ≤ D‐dimer	438	Yes	136	84	31	21	0.0083
		No	302	228	50	24	

a χ^2^‐test.

CAP group, described only D‐dimer values (copied and pasted from laboratory data); LDD group, listed differential diagnosis for positive D‐dimer; ND group, no description at all for positive D‐dimer; venous US, lower extremity venous US examination.

To address which factors had stronger effects for the outcome, we used the multivariate logistic analyses to examine the association of exposure (quality of the EMR documentation) with the outcome, adjusting for age, sex, value of D‐dimer, death on hospitalization, and primary disease (Table 4). Venous US was frequently carried out for patients assessed as having pulmonary embolism or DVT in the ED (OR 10.74; 95% CI, 5.92–19.50), surprisingly, even in the CAP documentation (OR 1.68; 95% CI, 1.20–2.40). As D‐dimer increased, venous US was carried out more often during admission with the OR increasing from 2.12 (95% CI, 1.46–3.02) to 2.92 (95% CI, 2.01–4.25) for D‐dimers of 3.0–10.0 and >10.0, respectively, although the effects of D‐dimer values were weaker compared to the high quality of the documentation (Table 4).

**Table 4:** Factors that influenced whether venous ultrasound (US) was undertaken during hospitalization following a positive D‐dimer measurement (≥1.0 μg/mL) that was taken in the emergency department (ED)

	Total 1,710 cases	Total venous US† 305 cases (17.84%)	Odds ratio (95% CI)	*P*‐value
Sex				
Female	782	182 (23.27)	1.00 (Ref.)	0.0033*
Male	928	123 (13.25)	0.65 (0.49–0.87)	
Age, years				
<80	967	131 (13.55)	1.00 (Ref.)	6.70E^−5^ *
≥80	743	174 (23.42)	1.78 (1.34–2.37)	
D‐dimer, μg/mL				
1.0 ≤ D‐dimer < 3.0	716	64 (8.94)	1.00 (Ref.)	
3.0 ≤ D‐dimer < 10.0	556	105 (18.88)	2.12 (1.46–3.02)	3.36E^−5^ *
10.0 ≤ D‐dimer	438	136 (31.05)	2.92 (2.01–4.25)	1.53E^−8^ *
Admission on emergency duty day				
No	712	185 (25.98)	1.00 (Ref.)	0.16
Yes	998	120 (12.02)	0.82 (0.62–1.08)	
Death in hospital				
No	1,431	278 (19.43)	1.00 (Ref.)	
Yes	279	27 (9.68)	0.42 (0.26–0.66)	0.0002*
Quality of the assessment at ED				
ND group	1,268	192 (15.14)	1.00 (Ref.)	
CAP group	332	64 (19.28)	1.68 (1.20–2.40)	0.0033*
LDD group	110	49 (45.54)		
DVT or pulmonary embolism		38 (35.32)	10.74 (5.92–19.50)	6.18E^−15^ *
Aortic dissection		8 (7.43)	3.45 (1.42–8.37)	0.0061*
DIC		3 (2.79)	1.31 (0.36–4.73)	0.68
Primary disease‡				
Cancer bearing	199	17 (8.54)	0.71 (0.41–1.25)	0.24
Infection	660	92 (13.94)	1.08 (0.77–1.50)	0.66
Trauma	343	131 (38.20)	3.72 (2.60–5.33)	9.59E^−13^ *
Others	591	76 (12.86)	1.00 (Ref.)	

^†^Data shown as *n* (%).

^‡^Multiple chosen.

The multivariate analysis was performed using logistic regression analysis. *Two‐sided *P*‐values <0.05.

CAP group, described only D‐dimer values (copied and pasted from laboratory data); LDD group, listed differential diagnosis for positive D‐dimer; DIC, disseminated intravascular coagulopathy; DVT, deep venous thrombosis; ND group, no description at all for positive D‐dimer; Ref., reference; venous US, lower extremity venous US examination.

### Factors influencing positive DVT findings

Finally, we reviewed the factors related to a final diagnosis of DVT. The 306 cases in whom venous USs were carried out summarize the results of multivariate logistic regression analysis, showing that the quality of documentation was key with an OR of 3.03 (*P* < 0.01) for the CAP group and 4.06 (*P* < 0.01) for the LDD group (listing suspected DVT or pulmonary embolism). However, the D‐dimer value was not identified as a significant factor with ORs of 1.24 (95% CI, 0.48–3.22) and 2.31 (95% CI, 0.91–5.91) for D‐dimers of 3.0–10.0 and >10.0, respectively (Table 5).

**Table 5:** Factors influencing positive deep venous thrombosis (DVT) findings in patients hospitalized following a positive D‐dimer measurement (≥1.0 μg/mL) that was taken in the emergency department (ED)

	Detected DVT from venous US 61 cases (19.9%)	Odds ratio	*P*‐value
Sex			
Female	39	1.00 (Ref.)	
Male	22	0.75 (0.39–1.46)	0.3900
Age			
<80	23	1.00 (Ref.)	
≥80	38	1.33 (0.69–2.55)	0.4000
D‐dimer (μg/mL)			
1.0 ≤ D‐dimer < 3.0	9	1.00 (Ref.)	
3.0 ≤ D‐dimer < 10.0	19	1.24 (0.48–3.22)	0.6600
10.0 ≤ D‐dimer	33	2.31 (0.91–5.91)	0.0800
Admission on emergency duty day			
No	39	1.00 (Ref.)	
Yes	22	0.63 (0.33–1.21)	0.1700
Death in hospital			
No	52	1.00 (Ref.)	
Yes	9	1.77 (0.67–4.67)	0.2500
Quality of the assessment at ED			
ND group	28	1.00 (Ref.)	
CAP group	17	3.03 (1.48–6.20)	0.0025*
LDD group (DVT or pulmonary embolism)	16	4.06 (1.75–9.46)	0.0011*
Primary disease†			
Cancer bearing	7	1.92 (0.61–5.99)	0.2600
Infection	13	0.32 (0.14–0.71)	0.0048*
Trauma	19	0.38 (0.17–0.83)	0.0160*
Others	22	1.00 (Ref.)	

^†^Multiple chosen.

The multivariate analysis was carried out using logistic regression analysis. *Two‐sided *P*‐values <0.05.

CAP group, described only D‐dimer values (copied and pasted from laboratory data); LDD group, listed differential diagnosis for positive D‐dimer; DIC, disseminated intravascular coagulopathy; ND group, no description at all for positive D‐dimer; Ref., reference; venous US, lower extremity venous US examination.

## Discussion

Our results suggest that high‐quality documentation by emergency physicians that describes the differential diagnosis for positive D‐dimer results on the EMR should have a greater influence than poor documentation that only describes the D‐dimer value (i.e., copy and paste from laboratory examinations) or that fail to provide any information. The precise description of the patients’ conditions using the EMR might improve the quality of medical care.

D‐dimer has high sensitivity and low specificity for DVT.^16^ Once a positive D‐dimer is identified in the ED, an ED physician should assess the reason for this increase, such as DVT, pulmonary embolism, or aortic dissection, and form a differential diagnosis. If this information can be effectively transmitted from emergency physicians to attending physicians through the EMR, venous US may be more readily undertaken by attending physicians.

Theoretically, the D‐dimer should be used only to exclude acute venous thrombosis, pulmonary embolism,^16^ aortic dissection,^11^ or other diseases.^12^ Therefore, in this study, all cases were equally considered “rule‐in” cases that were further evaluated for the “likelihood” of D‐dimer‐related diseases, including DVT. However, the actual D‐dimer value influenced the likelihood of performing a venous US during hospitalization (Table 3). Before ordering a D‐dimer examination, emergency physicians should consider the patients’ risk.^17^

Certain limitations of this study should be considered. First, its retrospective nature and the involvement of a single institution are important considerations. However, our exposure group setting included those without D‐dimer assessments; therefore, randomized control trials may not be suitable for ethical reasons, whereas information bias could be an obstacle in prospective observational studies.

An important consideration is that the attending physicians in the hospital may request a venous US based on the results of their own physical examination of patients, rather than relying on the information within the EMRs. However, in our system, the EMRs facilitate easy access to the ED records through electronic tags attached to the emergency physicians’ documentation. Therefore, all of the hospital‐attending physicians would refer to the patients’ EMRs written by emergency physicians. Our hospital survey reveals that 96.0% of the attending physicians referred to the documentation of emergency physicians before attending to the patient. Another confounding factor may be the length of hospital stay. According to the Wells score,^18^ patients bedridden for 3 days or more are considered at risk of DVT. However, although we measured the length of time from admission to the venous US (Table 1), we could not differentiate between the three groups (Tukey–Kramer method, *P* = 0.71).

This study provided the meaningful implications on the importance of the assessment at the ED. Indeed, EMRs support systems have been shown, in certain settings and for certain problems, to be associated with improved quality of care.^19^ In future studies, we should investigate how to better describe patients’ records on EMRs.

## Conclusion

Better documentation by emergency physicians regarding patients with positive D‐dimer results strongly affected the decisions made by the hospital‐attending physicians to order venous US, which was carried out even if the assessment only included copied and pasted information.

## Disclosure

Conflict of Interest: None declared.

## References

[1] Embi PJ , Yackel TR , Logan JR *et al* Impacts of computerized physician documentation in a teaching hospital: perceptions of faculty and resident physicians. J. Am. Med. Inform. Assoc. 2004; 11: 300–9. 15064287 10.1197/jamia.M1525 PMC436079

[2] Han H , Lopp L . Writing and reading in the electronic health record: an entirely new world. Med. Educ. Online 2013; 18: 1–7. 10.3402/meo.v18i0.18634 PMC3566375 23394976

[3] O'Donnell HC , Kaushal R , Barron Y *et al* Physicians’ attitudes towards copy and pasting in electronic note writing. J. Gen. Intern. Med. 2009; 24: 63–8. 18998191 10.1007/s11606-008-0843-2 PMC2607489

[4] Thielke S , Hammond K , Helbig S . Copying and pasting of examinations within the electronic medical record. Int. J. Med. Inform. 2007; 76(Suppl 1): S122–8. 16899403 10.1016/j.ijmedinf.2006.06.004

[5] Weis JM , Levy PC . Copy, paste, and cloned notes in electronic health records: prevalence, benefits, risks, and best practice recommendations. Chest 2014; 145: 632–8. 10.1378/chest.13-0886 24590024

[6] Geersing GJ , Zuithoff NP , Kearon C *et al* Exclusion of deep vein thrombosis using the Wells rule in clinically important subgroups: individual patient data meta‐analysis. BMJ 2014; 348: g1340. 24615063 10.1136/bmj.g1340 PMC3948465

[7] Rodelo JR , De la Rosa G , Valencia ML *et al* D‐dimer is a significant prognostic factor in patients with suspected infection and sepsis. Am. J. Emerg. Med. 2012; 30: 1991–9. 22795996 10.1016/j.ajem.2012.04.033

[8] Hagiwara S , Oshima K , Aoki M *et al* Usefulness of fibrin degradation products and d‐dimer levels as biomarkers that reflect the severity of trauma. J. Trauma Acute Care Surg. 2013; 74: 1275–8. 23609278 10.1097/TA.0b013e31828cc967

[9] Matsumoto Y , Yamabe S , Ideta K *et al* Impact of use of combined oral contraceptive pill on the quality of life of Japanese women. J. Obstet Gynaecol. Res. 2007; 33: 529–35. 17688623 10.1111/j.1447-0756.2007.00581.x

[10] Tovey C , Wyatt S . Diagnosis, investigation, and management of deep vein thrombosis. BMJ 2003; 326: 1180–4. 12775619 10.1136/bmj.326.7400.1180 PMC1126050

[11] Sutherland A , Escano J , Coon TP . D‐dimer as the sole screening test for acute aortic dissection: a review of the literature. Ann. Emerg. Med. 2008; 52: 339–43. 18819176 10.1016/j.annemergmed.2007.12.026

[12] Sathe PM , Patwa UD . D Dimer in acute care. Int. J. Crit. Illn. Inj. Sci. 2014; 4: 229–32. 25337485 10.4103/2229-5151.141435 PMC4200549

[13] Kahn SR , Ginsberg JS . Relationship between deep venous thrombosis and the postthrombotic syndrome. Arch. Intern. Med. 2004; 164: 17–26. 14718318 10.1001/archinte.164.1.17

[14] Nam JM . A simple approximation for calculating sample sizes for detecting linear trend in proportions. Biometrics 1987; 43: 701–5. 3663825

[15] Dentali F , Ageno W , Rancan E *et al* Seasonal and monthly variability in the incidence of venous thromboembolism. A systematic review and a meta‐analysis of the literature. Thromb. Haemost. 2011; 106: 439–47. 21725580 10.1160/TH11-02-0116

[16] Linkins LA , Bates SM , Lang E *et al* Selective D‐dimer testing for diagnosis of a first suspected episode of deep venous thrombosis: a randomized trial. Ann. Intern. Med. 2013; 158: 93–100. 23318311 10.7326/0003-4819-158-2-201301150-00003

[17] Pulivarthi S , Gurram MK . Effectiveness of d‐dimer as a screening test for venous thromboembolism: an update. N. Am. J. Med. Sci. 2014; 6: 491–9. 25489560 10.4103/1947-2714.143278 PMC4215485

[18] Wells PS , Anderson DR , Rodger M *et al* Evaluation of D‐dimer in the diagnosis of suspected deep‐vein thrombosis. N. Engl. J. Med. 2003; 349: 1227–35. 14507948 10.1056/NEJMoa023153

[19] Tamblyn R , Ernst P , Winslade N *et al* Evaluating the impact of an integrated computer‐based decision support with person‐centered analytics for the management of asthma in primary care: a randomized controlled trial. J. Am. Med. Inform. Assoc. 2015; 22: 773–83. 25670755 10.1093/jamia/ocu009 PMC4482273

